# Influence of sexual appeal in roadside advertising on drivers’ attention and driving behavior

**DOI:** 10.1371/journal.pone.0216919

**Published:** 2019-05-16

**Authors:** Norbert Maliszewski, Anna Olejniczak-Serowiec, Justyna Harasimczuk

**Affiliations:** 1 Institute of Psychology, Cardinal Stefan Wyszynski University in Warsaw, Warsaw, Poland; 2 Faculty of Psychology, University of Warsaw, Warsaw, Poland; Universitat de Valencia, SPAIN

## Abstract

Sexual appeals are widely used in advertising to attract consumers’ attention. It has already been proved that they influence the addressee’s cognitive processing, which in turn raises the question if sexual appeals may pose a serious threat for road safety when used in roadside advertising. Three studies were designed to answer this question. Study I was a nationwide survey (N = 1095) which revealed that drivers subjectively perceive sexual contents in roadside advertising as distracting and dangerous. Study II was a modified version of the Attentional Network Test (N = 1063) which proved that in cognitive tasks reaction time increases in line with the sexual content of advertisements. Study III was a simulator study (N = 55) which confirmed that driving characteristics change when sexually-oriented advertisements are located along the road. These studies have led us to a conclusion that sexually appealing cues in roadside advertising may pose a threat for road safety.

## Introduction

Sexual appeals are broadly defined as messages, either brand information in advertising context or persuasive appeals in social marketing contexts, that are associated with sexual information. Usually represented as images, verbal elements, or both, sexual information can be integrated with the message to a greater or lesser degree. Advertising research reveals that sexual appeals are attention-getting, arousing, affect-inducing, and memorable [1]. The aim of the present studies was to investigate the influence of sexual appeals in roadside advertising on drivers’ attention and behavior. Advertisements that attract the attention of drivers might be beneficial for advertisers but, at the same time, prejudicial to road safety. It seems crucial to understand the way in which sexual appeals in roadside advertising affect drivers, in order to limit such elements in situations where they may be dangerous for road safety.

### Influence of roadside advertising on drivers’ cognitive processes and behavior

The question of the influence of roadside advertising on road safety and drivers’ behavior emerged in the 1950s. Driver distraction/inattention have been identified as a major cause of road crashes [2], and many statistical analyses have proved that traffic accidents caused by driver inattention, are much more frequent in the presence of roadside advertisements [3]. Although some correlations between the presence of roadside advertising and road incidents had already been found, more in-depth studies comparing incidents on the same roads with and without advertising billboards installed, made the picture less clear [3–5].

Smiley [6] undertook a more detailed analysis of the influence of roadside advertising on Toronto traffic, and found out that even if no crashes occurred, certain driving characteristics (i.e. traffic flow, speed variance) were significantly affected by the presence of advertising billboards. Smiley’s findings suggest that what is crucial is not only the final effect of on-road events, i.e. a crash, but also a detailed analysis of drivers’ cognitive processes and behavior.

Even the first attempts to verify experimentally how cognitive processes are affected by advertising have consistently shown that when a driver is exposed to advertisements, their reaction time increases, and that the more advertisements are presented, the longer the reaction time [3]. A more recent study conducted on German highways [7] proved that when the number of supplementary signs increases, the driver’s ability to detect differences between the traffic signs decreases. Simulator studies by Bendak and Al-Saleh [8] performed on male drivers led to similar conclusions. Although no crashes occurred in either of the conditions (with vs. without roadside advertisements), driving characteristics were affected, i.e.: tailgating, overspeeding, drifting from lane, and crossing dangerous intersections recklessly were more common in the advertisement present condition, though only the differences concerning drifting from lane and reckless intersections crossing reached the level of statistical significance.

Apart from the driving characteristics, i.e. the number of crashes and lane deviations, Young et al. [9] took into consideration the cognitive load imposed on drivers by roadside advertising and proved that in the context of roadside advertising, drivers recalled fewer details of the route after the simulation and experienced higher cognitive load (as measured with NASA-TLX). The increase in subjective cognitive workload in the context of roadside advertising was also observed by Edquist et al. [10]. Their analyses also revealed longer reaction time (i.e. reaction to traffic signs) and higher visual dispersion. Horberry [11], on the other hand, observed some decrease in vehicle control in the context of roadside advertising, consisting of lower speed and weaker reaction to danger (operationalized as the lowest speed obtained in reaction to a threat, e.g., a pedestrian entering the road). Interestingly, both Horberry and Edquist [10–11]) concluded that the effect of roadside advertising was stronger in older drivers.

To sum up, although the direct impact of roadside advertising on the number of accidents in not always visible in research, it is clearly evident that the presence of roadside advertisements leads to crucial changes in the driver’s behavior when both driving behavior and cognitive resources consumption are considered. A new question emerges here, namely: does the advertising content relate to its influence on the driver?

### Influence of roadside advertising content on the road safety

The problem of the relation between the content of roadside advertising and the influence that it has on the driver’s behavior and road safety remains less investigated. The study performed by Kaber [12] proves that information signs which include logotypes of service points attract more attention than regular traffic signs, being at the same time more distracting. Moreover, Zhang and her team [13] proved that the distractive value of an information sign increases with the number of logotypes shown. There is also some evidence available that a large amount of textual information included in an advertisement results in driver distraction [14].

Nonetheless, the actual content (the essence, emotional load) of an advertisement is not a widely researched topic. According to Chan and Singhal [15], emotionally loaded billboards affect driver behavior, though the influence depends on the valence of the emotional stimuli. In the simulated driving study, negative emotional stimuli caused lane departures and slowing down, while positive–lane departures and speeding. Studies by Megías [16–17] reveal distractive value of negative emotional load of advertisements resulting, among others, in an increase of speeding in reaction to yellow traffic light (as compared to positive emotional load). On the other hand, the study presented by Olejniczak-Serowiec [18] suggests negative influence of positive emotional load in advertisements on attention and performance in cognitive tasks, especially when attention to peripherally presented stimuli is considered.

### Influence of sexual appeals on cognitive processing

Although, to our best knowledge, there are no studies on the influence of sexual contents in roadside advertising on the drivers’ attention and behavior, sexual appeals in advertising as such are an issue represented quite well in the literature. Studies have demonstrated that sexual appeals direct one’s attention towards the advertisement, typically without any corresponding advantage for brand processing (e.g., brand name recalling; [19–23]. Findings that deny advantages for brand processing despite the attention catching capacity of sexually appealing advertisements [24] resulted in advancing the proposition that hedonic appeals (i.e. sex) increase motivation to process the advertisement content, largely at the expense of the brand. Moreover, some studies prove that sexual advertisements cause arousal and positive emotions [25–26].

Notwithstanding the effect of sexual advertisements on brand processing, it remains clear that sexually appealing contents attract attention and engage it in the advertisement execution, supposedly diverting it from the road if the advertisement is placed on the roadside. Studies on the influence of secondary tasks on driving behavior prove that attention engagement in secondary tasks reduces driving effectiveness, especially when visual attention is taken into consideration [27–31](.

### Current study

Studies on the psychology of advertising point out that sexual appeals catch attention, but that attention is focused on sexual stimuli, while brand-related information remains neglected. The question can be raised whether sexual appeals in roadside advertising attract drivers’ attention, leading to disregarding other stimuli, including those critical for safe driving. This would mean that sexual stimuli are so appealing that they divert attention from the road for such a long time that the driver fails to notice emerging danger or loses situational awareness. It is assumed that looking away from the road for 2 seconds constitutes a hazard to driving safety [32]. For example, exposition to long advertising slogans may cause the driver to look away from the road for a long time and make rapid movements with the steering wheel to return to the correct position in the lane. However, sexual stimuli, based on pictures, not on text, is not subject to linear processing. Images are processed holistically, and their meaning can be processed by human brain even if seen as briefly as 13 milliseconds [33]. This means, that even if the driver takes only a short look at sexually appealing advertisement, it may be processed in his mind much longer. Furthermore, apart from diverting one’s attention, sexual appeals cause arousal and emotions which can affect the driver’s attention management.

Below a series of three studies is presented. All of them are focused on the influence of sexually appealing roadside advertising on drivers functioning. To date, the question is more present in media than in scientific journals. In 1990’s billboards advertising underwear in Norway proved so distracting to drivers, that they have been banned. Similarly, in 2014, in Milan, Italy, the Police had to remove sensual underwear billboards to restore traffic safety [34]. Peculiar PR action in Moscow, Russia, in 2014, in which trucks with naked female breasts were driving through the city, resulted in 517 crashes in a day [35]. Such information make it clear, that sexual advertising might pose a serious threat to traffic safety, however, not much research results can be found in the domain. The first study was designed to verify whether roadside advertising is socially perceived to be a safety related issue (research question 1), and to verify whether advertisements with sexually appealing contents are subjectively perceived, by the drivers, as attention-diverting more frequently than other types of contents (research question 2). The second was designed to find cognitive mechanism underlying the distraction experienced by the drivers (research question 3). Our last, third study, aims at verifying how this mechanism influences driving behavior (research question 4).

In the current study, advertisements with sexual stimuli of various intensity were used. We hypothesized that more intense sexual stimuli would draw the driver’s attention and affect their driving behavior more strongly. In the first study, there are two hypotheses. First, we hypothesize, that roadside advertising is perceived by the drivers as a hazard to road safety (hypothesis 1a). Secondly, we hypothesize that it’s not negative stimuli (as suggested by the aforementioned studies), but sheer emotional arousal that is perceived to be distractive by the drivers. Briefly speaking, we hypothesize that sexual advertisements are subjectively perceived by the drivers as more distractive than any other type of advertising content, negative content excluded (hypothesis1b). In study two, we test a hypothesis that sexually appealing advertisements affect attention management more than other types of contents (hypothesis 2a), and that advertisements with more intense sexual stimuli cause a decrease in attention management (hypothesis 2b). To verify this hypothesis, a modified version of the Attentional Network Test [36], named brief-ANT [37], was used. In the third study, a hypothesis that attentional engagement in sexual stimuli disrupts driving behavior (hypothesis 3a) was tested using a driving simulator, and that the higher level of sexual appeal, the stronger the influence on driving behavior (hypothesis 3b).

We conducted the studies in conformity with the Code of Ethics of the Polish Psychological Association and in accordance with the ethical standards as laid down in the 1964 Declaration of Helsinki and its later amendments. The experimental protocol was approved by the Faculty of Psychology, University of Warsaw ethical committee (the whole procedure was opinioned, including: recruitment, methods, instructions, informed consent, information provided to participant, etc.) and the methods were carried out in accordance with the approved guidelines. Written/oral informed consent was obtained from each subject/participant prior to the study.

All the studies were conveyed as a part of a larger research project on the influence of roadside advertisement on driving safety funded by the National Centre for Research and Development and the General Director for National Roads and Motorways (DZP/RID/I33/4/NCBR/2016).

## Study I

### Participants and procedure

The study was conveyed on a quota sample of drivers representative for the population of Polish drivers with regard to sex, age, and place of living according to the statistical data [38]. 1095 Polish drivers were involved in the study, 428 female and 667 male. The youngest participant was 18 and the oldest was 80 (*M* = 41.43; *SD* = 14.29). All respondents had been active drivers for up to 51 years (*M* = 16.77; *SD* = 11.56). 66% of respondents declared using a car on daily basis, and 21%–at least once a week (the rest of the group declared using their car less frequently). Study I was a nationwide CAWI (Computer-Assisted Web Interview) research in which the participants filled in an online survey. All participants were research panel’s voluntary respondents. The research panel sent invitations to take part in the study to email addresses collected from the owners of web portals with over 20 million users, and invited the users to participate in the study via email. The participants were invited to fill in the survey and express their opinion on the driving safety-related issues. The participants were informed that the study lasts about 15 minutes and that they will be rewarded with a certain amount of credits), and that the study is anonymous. The participants were rewarded for handing in the survey with the declared amount of credit points that they could subsequently exchange for rewards in the panel’s “rewards-shop” (each item has its’ price expressed as a number of credit points, and the respondent decides on exchanging his points for a reward after a single study, or to collect more credits by handing in more surveys; sample rewards are: books, cosmetics, kitchen appliances, etc.).

### Method and materials

First, each participant completed a survey on their demographics, driving experience and health. Next, they answered questions about their opinions and experiences concerning roadside advertising in general, and specific contents of roadside advertisements.

Respondents were asked to express their opinion on roadside advertising using a 5-point Likert scale (where 1 –*I disagree*, and 5 –*I agree*), on which they were to indicate to what extent they agreed with the following statements:

- *Roadside advertisements make roads less monotonous*.
- *Roadside advertisements’ influence on road safety is a serious social issue*.

Questions concerning general experience with advertisements were as follows:

- *Has it ever happened to you that a roadside advertisement distracted your attention while you were driving*?
- *How often does it happen that roadside advertisements distract your attention while you are driving*?
- *How often does it happen that roadside advertisements make it difficult for you to maintaining situational awareness while you are driving*?
- *In your opinion*, *how often may roadside advertisements affect anticipation of the situation on the road*?

In the final section participants were asked how often different types of advertising content distract their attention. The question was phrased as follows: *How often does it happen that sexual (emotionally negative*, *emotionally positive*, *humorous*, *intriguing*, *including sale offers) advertisements distract your attention while driving*?

In all questions concerning frequency, respondents answered on a scale where “0” meant *Never*, “1”–*Very rarely*, “2”–*Rather rarely*, “3”–*Neither rarely*, *nor often*, “4”–*Rather often*¸”5”–*Very often*, “6”–*Always*. “*I don’t know”* was a possible answer as well. Each question contained a picture illustrating an example of the type of advertisement content in question; the illustrations were photos of real advertising billboard. For each type of advertisement, two pictures were chosen in order to exclude possible influence of brand familiarity on the study results. The choice was preceded with a pilot study on 30 subjects. On the basis of the results, advertisements which represented each type/technique best were chosen.

### Results

The data were analyzed using IBM SPSS Statistics; in all the analyses, answer *7*: *I don’t know* was recoded into *missing values*.

The analysis of the opinion-based questions revealed that the mean score for answers on the positive assessment scale (*M* = 2.6, *SD* = 1.07) was significantly lower than the one on the negative assessment scale (*M* = 3.61, *SD* = 1.04; *t* = -19,919, *p < 0*.*001*, *d = 0*.*953)*.

Frequency analysis of the first experience-related question (*Has it ever happened to you that a roadside advertisement distracted your attention while you were driving*?) revealed that 42.8% of respondents experienced distraction by roadside advertising. The next three questions were only presented to these 42.8% of respondents. The results of frequency analyses for the remaining questions on general experience with roadside advertisements are presented in Table 1.

**Table 1:** Frequency analysis for questions related to experiences with roadside advertisements.

Answer	*n*	Percent of the answers
*How often does it happen that roadside advertisements distract your attention while you are driving*?		
Never	2	0.4
Very rarely	56	12.2
Rather rarely	114	24.8
Neither rarely, nor often	115	25
Rather often	119	25.9
Very often	42	9.1
Always	12	2.6
*How often does it happen that roadside advertisements make it difficult for you to maintaining situational awareness while you are driving*?		
Never	32	7
Very rarely	70	15.4
Rather rarely	105	23
Neither rarely, nor often	110	24.1
Rather often	98	21.5
Very often	37	8.1
Always	4	0.9
*In your opinion*, *how often may roadside advertisements affect anticipation of the situation on the road*?		
Never	3	0.7
Very rarely	13	2.8
Rather rarely	37	8.1
Neither rarely, nor often	84	18.4
Rather often	181	39.6
Very often	107	23.4
Always	4	0.9

Descriptive statistics related to the declared frequency of being distracted by particular types of roadside advertising content are presented in Table 2.

**Table 2:** Roadside advertisement content and the frequency of being distracted: descriptive statistics.

Type of contents	*M*	*SD*	*%* *Rather often*, *very often*, *always*
Sexual	2.89	1.67	36.1
Positive	2.47	1.44	22.8
Negative	2.89	1.56	37.8
Humorous	2.68	1.49	28.7
Sale offers	2.38	1.54	23.8
Intriguing	2.88	1.55	36.9

The answers to the questions about distraction from positive, negative, humorous, intriguing and sales offerings were averaged, and the obtained value (*M* = 2.66, *SD* = 1.31) is used as a reference to assess sexually appealing contents distractiveness. The difference was statistically significant (*t* = -6.441, *p* < 0.001) with the mean distractiveness of sexual contents assessed higher (*M* = 2.89, *SD* = 1.67) than the averaged assessment of other types of contents.

**Fig 1:**
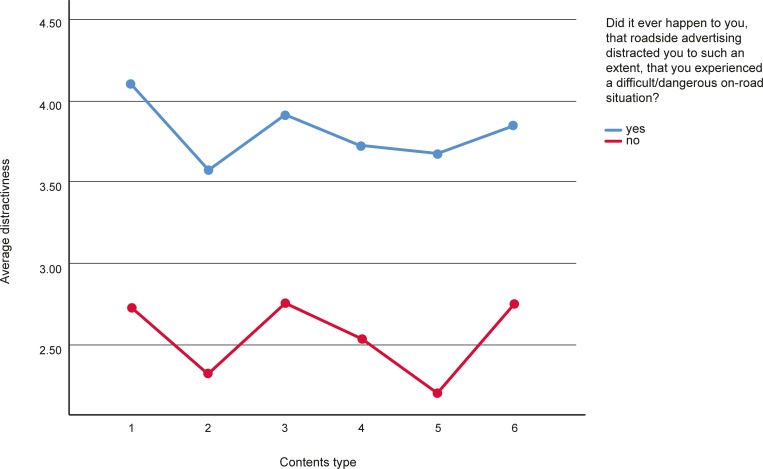
Interaction effect of content type and experience of difficult/dangerous on-road situation due to distraction from roadside advertisement. Types of contents: 1- Sexual, 2- Positive, 3- Negative, 4- Humorous, 5- Sales offers, 6- Intriguing.

General linear model analysis with the Greenhouse-Geisser test revealed significant differences between the declared frequency of being distracted by different types of content (*F* = 67.51; *df* = 4.649; *p* < 0.001; η^2^ = 0.061). Contrast analysis shows that sexual content is significantly more distracting than positive content (*F* = 96.426; *df* = 1; *p* < 0.001; η^2^ = 0.086), humorous content (*F* = 30.579; *df* = 1; *p* < 0.001; η^2^ = 0.029), or sale offers (*F* = 133.517; *df* = 1; *p* < 0.001; η^2^ = 0.115). The differences between sexual and negative or intriguing content were not significant (*F* = 0.002; *df* = 1; *p* = 0.965; η^2^ = 0.000 and *F* = 0.0559; *df* = 1; *p* = 0.809; η^2^ = 0.00). Interestingly, interaction effect between the type of contents and experience of a difficult/dangerous on–road situation due to distraction from advertising was observed (*F* = 2.718; df = 4.649; p = 0.021). 12% of respondents declared that they had experienced difficult/dangerous on-road situation due to distraction from roadside advertising. Contrast analysis revealed, that the interaction effect was statistically significant (*p* = 0.022) for one pair of contntents type only, namely, those who had experienced difficult/dangerous situation due to distraction from roadside advertising perceived sexual advertising as more distractive (*M* = 4.06, *SD* = 1.53) than those with intriguing contents (*M* = 3.84, *SD* = 1.38). For other types of contents, there were no significant differences (see Fig 1).

**Fig 2:**
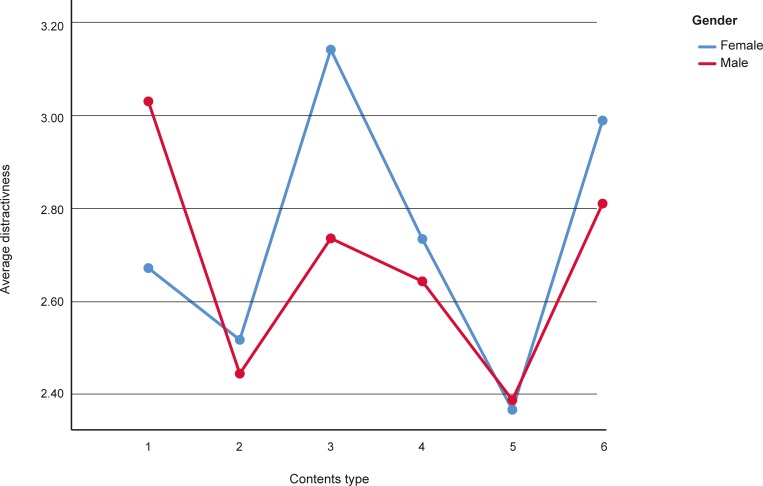
Interaction effect of content type and gender. Types of contents: 1- Sexual, 2- Positive, 3- Negative, 4- Humorous, 5- Sales offers, 6- Intriguing.

Additional analyses concerning demographical data revealed that men perceive sexual contents in roadside advertisements as more distractive than women (t = -3.324, p = 0.001; M = 3.02, SD = 1.65; M = 2.67, SD = 1.67 respectively). Interaction effect between contents type and gender was also statistically significant (*F* = 19.764; df = 4.672; p < 0.001; see Fig 2). Men perceived sexual advertising as more distractive than any other type of contents (*ps* < 0.001).

Also, the age groups differed significantly with regard to the perceived distractiveness of sexual advertisements (F = 3.385; p = 0.009; η^2^ = 0.013). Post hoc analysis with Bonferrioni test revealed, that the oldest group (> 54 years; M = 2.58, SD = 1.67) assessed sexual advertisements as significantly less distracting than the youngest group (18–24 years; M = 3.15, SD = 1.78, p = 0.016), and those between 25 and 34 years old (M = 2.87, SD = 1.60; p = 0.023). No other differences between age groups were significant (p > 0.05). No significant interaction between sex and age was observed (F = 0.078, p = 0.989).

Neither the frequency of driving (F = 0.196, p = 0.978) nor the driver’s average yearly mileage (F = 0.200, p = 0.977) proved to be a significant factor in the analysis.

### Discussion

The results of Study I have proved that roadside advertising and its influence on road safety is a socially perceived hazard (hypothesis 1a). It has also shown that almost half of the drivers report distraction due to roadside advertising. Many of them have also reported more or less frequent attention problems while driving in the presence of advertisements. Finally, respondents placed sexual advertisements among those most distracting, with distraction frequency comparable to negative and intriguing contents (hypothesis 1b) in general, while for male respondents, sexual advertising is clearly the most distractive type.

The results of Study I, led us to a conclusion that distraction by sexual advertisements is an important issue, worth exploring and formulating research questions about the influence of sexual roadside advertising on drivers’ attentional processes and driving behavior. Consequently, Study II was designed to search for cognitive mechanism underlying the perceived distraction from sexual advertisements.

## Study II

### Participants and procedure

A representative sample of 1063 Polish drivers were involved in the study, 404 female and 659 male. Similar to Study I, a quota sample was used to reflect the Polish drivers population. The youngest participant was 18 and the oldest was 77 (*M* = 41.37, *SD* = 14.00). 66% of the respondents declared using a car daily, and 21%–at least once a week (the rest of the group declared using a car less frequently). Study II was a nationwide CAWI research in which the participants filled in an online survey. The survey contained Brief-ANT (the modified version of ANT test described below) and a number of survey questions to collect demographic data. Recruitment procedure was identical with the one described in Study I.

### Methods and materials

Each participant filled in a demographic survey, followed by a modified version of the Attentional Network Test [36]. The aim of the research, unlike in the original Attentional Network Test, was to examine the attention management system (rather than the three aspects of attention proposed by [39]). As a result, we developed a short version of the test, based on the 10-minute version of the ANT developed by the CRSD [40], which will be referred to as brief-ANT hereinafter. This version of the test consists of 2 blocks of 64 trial sequences each (ordered randomly). The total number of 64 trials per block are organized in the following sequences: 2 (repetitions) x 4 (cue conditions: no cue/center cue/double cue/spatial cue) x 2 (flanker conditions: congruent vs. incongruent) x 2 (target positions: above or below) x 2 (target directions: right or left). Each trial consists of the following sequence (one for each cue condition): fixation (random* presentation time: 400ms to 1200ms) -> cue (100ms) -> fixation (400ms) -> target (1500ms)-> intertrial interval (3000ms).

**Fig 3:**
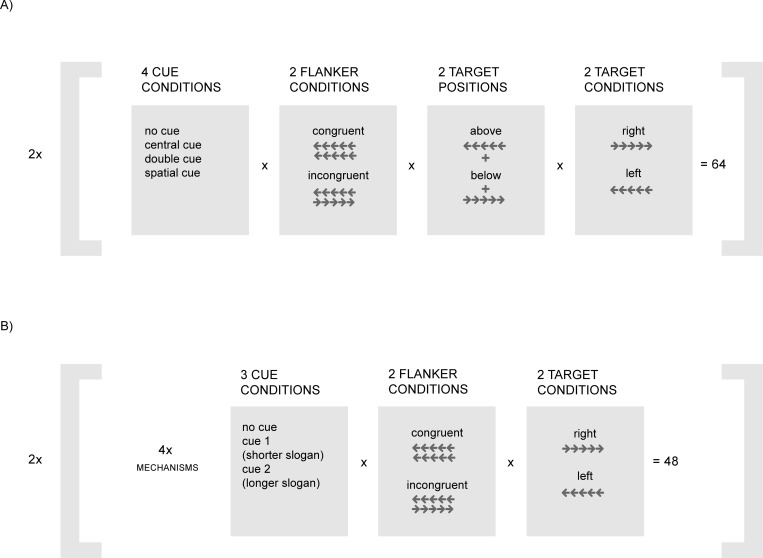
The sequence of screens in a trial in the original version of ANT (top) and in a trial in brief- ANT (bottom).

In brief-ANT, the fixation cue is either an advertisement (450x300px) or a cross (like in the original; cf. Fig 3). We set a constant presentation time: 2 seconds, the safety-critical time for visual attention distraction according to the transportation research. Then, the cue followed consistently with the original, but the re-fixation time was also changed, i.e. shortened to 100ms. This was done to reduce the time between exposition to the advertisement and reaction. We assumed that advertisements distracted drivers’ attention from the road, even though drivers should react quickly to the changing situation on the road. Finally, the target was presented for 1500ms. To sum up, the brief-ANT trial consisted of the following sequence: fixation (2000ms) -> cue (100ms) -> fixation (100ms) -> target (1500ms)-> intertrial interval (3000ms).

In the study, advertisements incorporating different types of content were used: four advertisements with sexual contents varying with regard to the intensity of the sexual cue (2 strong sexual cues and 2 weak sexual cues), and four reference (nonsexual) cues. The choice of advertisements used in the study was based on the results of a pilot study (N = 30). Ten pairs of advertisements varying with regard to the intensity of sexual cue were prepared, the same model was presented in each of the two pictures (e.g. wearing underwear or naked). For the proper study, pairs for which the pilot study had revealed differences in the level of sexual content but not in the overall attractiveness of the advertisement were chosen.

### Results

Due to missing data, analyses are presented for a sample consisting of 972 respondents. Descriptive statistics for reaction time and number of errors in the context of sexual (strong and weak) and nonsexual advertisements are presented in Table 3.

**Table 3:** Reaction time and number of errors in the context of sexual (strong and weak) and nonsexual advertisements.

Advertisement content	*M*	*SD*
*Reaction Time [ms]*		
Sexual	663.13	164.80
Strong	669.03	185.82
Weak	656.01	179.85
Nonsexual	651.91	161.32
*Errors*		
Sexual	0.16	0.30
Strong	0.16	0.33
Weak	0.16	0.33
Nonsexual	0.16	0.30

T-test for dependent samples proved that reaction times in the context of sexual advertisements were significantly longer than in the context of nonsexual advertisements (*t* = 3,144, *p = 0*.*001*, *d = 0*.*069)*. Analyses taking into account the level of sexual content in the advertisements show that the difference remains significant for highly sexual advertisements (*t* = 3,933, *p < 0*.*001*, *d = 0*.*097)*, while it disappears for weak sexual cues (*t* = 0,923, *p = 0*.*178*, *d = 0*.*024)*. The differences between reaction times for the two different levels of sexual cues were also significant (*t* = -2,544, *p = 0*.*006*, *d = 0*.*071)*; the stronger the sexual cue, the longer the reaction time. With regard to the number of errors, no differences were found.

Unlike in the case of subjective feelings, the cognitive test did not reveal any sex differences in reaction to sexual contents (p > 0.05). Interaction effect between content type and gender was also nonsignificant (*F* = 0.747; *p* = 0.474). Age group and average yearly mileage do not make any difference (*ps* > .05) either.

### Discussion

Although no differences were identified between sexual and nonsexual advertisements in terms of the number of errors, reaction time–one of the key variables in vehicle driving–was significantly affected. Reaction times were considerably longer for sexual advertisements, possibly due to the emotional affect or arousal still present in the subjects after the presentation of the cue (hypothesis 2a). Such a conclusion is further supported by the fact that the effect was only present in the context of highly sexual advertisements (hypothesis 2b). Lack of gender differences, suggests that despite the difference in subjective feelings, men and women are equally sensible to the influence of sexual advertising on attentional management.

Study III was designed to verify, how the influence of sexual advertising on attention management shows up in the real driving behavior.

## Study III

### Participants and procedure

Experimental sample consisted of 55 drivers (29 male and 26 female). All the participants had been active drivers for at least one year and had driven at least 3000 km in the preceding year. The youngest participant was 18 and the oldest was 64 (*M* = 38.71, *SD* = 15.41). 40% of respondents declared using their car daily, and 46%–more often than once a week (the rest of the group declared using their car less frequently). All the participants were volunteers recruited by a specialist research recruitment company and were paid for their participation in the study.

When the participants arrived at the laboratory, having signed a formal agreement to participate in the study, they were informed about the procedure and started their first simulator drive: an approx. 10-minute adaptation drive dedicated to getting acquainted with the simulator. Afterwards, they filled in a demographic survey. Then, a test drive and a control survey concerning their subjective feelings about the presented advertisements followed. After each drive, the participants were asked about their general condition, also during the entire simulation, participants were able to inform the researcher that their condition got worse or they wanted to withdraw from the study.

### Method and materials

#### Demographic survey

Participants were asked to answer a set of questions regarding their sex, age, frequency of driving a car, and driving-related experiences. We also checked their state of health (i.e. sight impairment).

#### Control survey

Participants were asked to fill in a survey, in which all the stimuli used in the simulator study were presented, each—followed by a set of questions concerning the content (e.g., *To what extent do you agree that*: *the advertisement is sexual*?). Each participant was asked to answer them on a 7-point Likert scale.

#### Driving simulator

The study was conducted using a professional Opel Astra-based AS1200-6 driving simulator belonging to the Motor Transport Institute in Warsaw, where the study took place. The simulator consists of a driving cabin placed on a moving platform with six degrees of freedom (angular movements: shift ±22°, speed ±30°/s, acceleration ±500°/s^2^; linear movements: shift ±0.25m, speed ±0.5m/s, acceleration ±0.6g), and a 60 Hz projector located 2.5 meters away from the driver’s eyes (the projector covers the visual field of 40°x200°).

**Fig 4:**
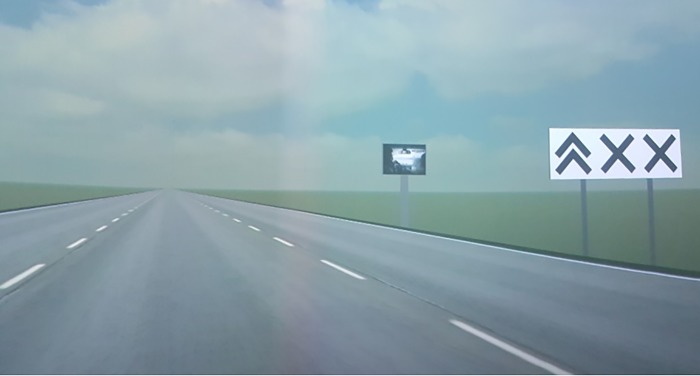
The modified LCT task [source: Own photo].

Driving scenario was prepared on the basis of a standardized *Lane Change Task* (LCT), one of the *controlled driving tasks* developed in the Driver Workload Metrics project for the OpenDS Pro platform [41]. During the test drive, the task of the driver was to drive a car as usual, and follow the traffic signs (which instructed them to change driving lanes). Each participant drove for approximately eight minutes on a three-lane non-urban (60 kph) road. The drive was divided into training part and the actual test part. Along the road, traffic signs as well as advertising billboards sized 6144x2560px were located. Their size related to the natural dimensions of 12x5m. The billboards were located 40m away from the road, and were visible from the distance of 140 m. Fig 4 presents the modified version of LCT task with an advertising billboard added.

Two billboards were devoid of sexual contents (control condition) and six billboards were sexually-oriented (divided into three levels of sexual appeals: weak, moderate and strong). Apart from sexual cues, the billboards also included names of widely known brands. The choice of advertisement used in the study was based on the results of a pilot study (*N* = 30). Ten triads of advertisements varying with regard to the intensity of the sexual cue were prepared, with the same model presented in each of the three pictures (e.g., wearing underwear, with her breasts covered, or totally naked). For the study, triads for which the pilot study had revealed differences in the level of sexual content, but not in the overall attractiveness of the advertisement were chosen. In the weak sexual cue, the model was seductive, but dressed up; in the moderate sexuality condition, the model was wearing lingerie; while in the strongly sexual version of the cue naked body was presented.

### Results

The manipulation control survey analysis confirmed that sexual cues used in the study were subjectively perceived as significantly more sexually appealing than any other cues (*t* = 24,51, *p < 0*.*001*, *d = 4*.*58)*. The mean results on the sexuality scale for sexual cues was 5.27 (*SD* = 0.16), while the mean for other cues was 1.15 (*SD* = 0.05).

Table 4 presents means and standard deviations for behavioral variables measured during the simulation drive, calculated as the mean value for all the sections where a sexual advertisement was presented and for all nonsexual advertisement sections.

**Table 4:** Means and standard deviations for behavioral variables measured during the simulation drive.

	Sexual advertisement								Nonsexual advertisement	
	Total		Weak		Moderate		Strong			
	*M*	*SD*	*M*	*SD*	*M*	*SD*	*M*	*SD*	*M*	*SD*
Reaction time	13.77	1.47	13.86	3.03	13.27	0.68	13.95	2.97	13.18	0.69
Speed	58.29	3.62	58.42	6.44	59.39	5.56	57.06	7.72	59.70	4.93
Speed SD	2.58	2.46	2.43	5.16	1.75	4.42	3.55	6.00	1.32	3.83

The t-test analysis for dependent samples revealed a significant difference between two conditions: one with a sexual advertisement with and one with a nonsexual advertisement, for the reaction time variable (i.e. time between the instruction to change lanes and the actual change; *t* = 2.719, *p = 0*.*005*, *d = 0*.*447)*. We also found statistically significant differences for speed (*t* = -1,792, *p* = 0.04, *d =* 0.325) and for the standard deviation of speed (*t* = 1.844, *p = 0*.*036*, *d = 0*.*392)*. No significant differences were found for different levels of sexual appeals.

Analysis with the use of demographic data (age and gender) did not reveal any statistically significant effects, there were no interaction effects either (*ps* > 0.05).

### Discussion

The two conditions: with and without sexual advertisements, differed significantly with regard to the driving characteristics (hypothesis 3a). Although it did not cause speeding (on the contrary: the mean speed was lower in the sexual condition), the fluency of driving was disrupted (i.e. the standard deviation of speed increased in the sexual condition). Moreover, the time between a lane change cue and the actual lane change increased significantly in the sexual condition. No effects of sexual cue intensity were observed (contrary to hypothesis 3b). It is possible that differences in the sexual cue intensity were well recognized in the subjective-declarative assessment (pilot study) where models differed with regard to their clothing; however, naked models did not necessarily provoke stronger sexual reactions (when arousal is considered rather than declarations), meaning that the opposite may even be the case. The results confirm the influence of sexual advertisements on driving behavior, whereas the question of the intensity of sexual cues requires further research.

## General discussion

The poll study discussed as Study I confirmed that roadside advertising is an important social issue. Moreover, it revealed that the widely used advertising mechanism of attracting attention with the use of sexually appealing stimuli is subjectively assessed by Polish drivers, especially men, as one of the most distracting and potentially dangerous. Even more so, if they have ever fall in dangerous situation on the road, which they do associate with having been distracted by roadside advertisement.

Since proper attention management is widely acknowledged to be a key factor for road safety and maintaining situational awareness, Study II explored the influence of sexual cues on attention management. Consistently with our hypotheses, we observed that reaction time in the context of sexual advertisements was significantly longer than in the context of nonsexual stimuli. Although it did not affect the reaction accuracy, the authors conclude that sexual advertisements clearly affected attentional system. We suppose that longer reaction times resulted from attention being engaged elsewhere, outside the task. It could be that if we were to define a shorter time for reaction, the number of errors would increase accordingly. Interestingly, gender difference observed in the subjective assessment, did not reveal itself in Study II, which might mean that despite declarative differences, men and women are equally sensitive to the influence of sexually appealing advertisements on attention management.

If our conclusion about attention overload when exposed to sexually appealing stimuli is correct, then driving characteristics in the presence of a sexual roadside advertisement would change, especially when some kind of reaction is required from the driver. To simulate such a situation, a modified Lane Change Task was used in Study III. Its results show (consistently with hypothesis 3a) that driving characteristics change in the presence of sexually appealing billboards. Although speed as such was not found to increase (on the contrary, it decreased), driving fluency was clearly disrupted. It is possible that drivers actually slowed down while passing by a sexual advertisement to have a better view of it, which implies looking away from the road. Another, even more compelling explanation is that in the presence of a sexual advertisement, drivers’ attention was overloaded or engaged away from the road, leading to the driver losing control of the vehicle and its speed. This conclusion is further supported by the increase in the standard deviation of speed in the context of sexual advertisements. Finally, consistently with the results of Study II, an increase in reaction time–here operationalized as the time between the occurrence of lane change traffic sign and the actual lane change–was observed. Also in line with the results of Study II, no gender differences were found in the influence of sexual stimuli on driving behavior, which leads us to a conclusion that sexual stimuli in roadside advertising influence attentional management in men and women equally, and the influence results in disrupted driving fluency. Although the observed behavioral differences in driving caused by sexual contents and other types of contents were not dramatic, it should be clearly stated that traffic accidents are statistically rare occurrences resulting from multiple factors emerging at the same time. Consequently, no strong effects could be expected. Although accidents caused by sexual stimuli might be rare, overlooking this cause may pose serious, if not fatal, consequences. We hypothesize that behavioral changes may result from emotional arousal caused by sexual appeals in advertisements. Although emotional arousal is defined as one of the key factors enabling cognitive functioning (due to its energizing value; cf.: [42]), Kahneman [43] points out that strong emotional arousal may engage attention to such extent that there are not enough attentional resources left to handle the task at hand. Studies on the effectiveness of emotionally arousing advertisements, understood as buying the advertised product, show a correlation between the strength of emotional stimuli and behavior. As much as strong negative emotions may result in engaging attention and following the advertisement [44–47] or in stimulus avoidance [48], positive stimuli are often associated with mood regulation and a tendency to stay in a good mood [49], which may lead to a longer attention focus on the positive stimuli. Such an idea finds some support in our aforementioned previous study [18], where cognitive efficiency was found to decrease in the context of emotionally positive stimuli.
